# Health-related quality of life and willingness to pay measurement among patients on warfarin in Thailand

**DOI:** 10.1186/s40545-023-00632-2

**Published:** 2023-10-30

**Authors:** Nattanichcha Kulthanachairojana, Krittaphas Kangwanrattanakul, Thitiya Khongmee, Nampratai Pawasan, Sitanan Chityam, Suphannika Pornwattanakavee

**Affiliations:** 1 Division of Social and Administrative Pharmacy, Faculty of Pharmaceutical Sciences, Burapha University, 169 Long-Hard Bangsaen Rd., Mueang, Chonburi, 20131 Thailand; 2 Department of Pharmacy, Chonburi hospital, Chonburi, Thailand; 3 Department of Pharmacy, Faculty of Medicine, Burapha University hospital, Chonburi, Thailand; 4 Department of Pharmacy, Banglamung hospital, Chonburi, Thailand; 5 Division of Clinical Pharmacy, Faculty of Pharmaceutical Sciences, Burapha University, Chonburi, Thailand

**Keywords:** Health-related quality of life, Willingness to pay, Patients on warfarin, Thailand

## Abstract

**Background:**

Complex dosage regimens and the high incidence of adverse events associated with warfarin therapy can affect the health-related quality of life (HRQoL) and willingness to pay (WTP) among patients on warfarin. No such previous study has been conducted to assess the HRQoL and WTP among Thai patients on warfarin; therefore, this study aimed to measure these parameters and identify some sociodemographic factors associated with those aspects among patients on warfarin in Thailand.

**Methods:**

This cross-sectional survey study involving 260 patients on warfarin between June 2022 and June 2023 used a quantitative method for data collection. Face-to-face interviews with well-trained interviewers were conducted and patients were required to complete the questionnaires of both World Health Organization Quality of Life-BREF (WHOQOL-BREF) and EQ-5D-5L to assess and measure their HRQoL levels. WTP was assessed using a bidding game technique. Descriptive statistics with mean and standard deviations were used to report HRQoL scores and WTP, whereas a generalized linear model was employed to identify factors associated with both HRQoL and WTP.

**Results:**

The mean EQ-5D index and mean EQ-VAS score were 0.89** ± **0.15 and 76.92** ± **15.95, respectively, whereas the mean WHOQOL-BREF domain scores were 59.18 ± 14.13, 68.56 ± 15.47, 59.13 ± 19.64, and 65.23 ± 14.04 for the physical, psychological, social, and environmental domains, respectively. Elderly participants (age > 60 years) and those with comorbidities had lower HRQoL scores than their counterparts. The mean WTP was 22.25** ± **32.19 USD for one patient’s visit. The presence of comorbidities was the only factor significantly associated with WTP values.

**Conclusions:**

Thai patients on warfarin have lower mean EQ-5D indexes and EQ-VAS scores than members of the general Thai population. Patients on warfarin with comorbidities have diminished HRQoL and WTP values. Therefore, all healthcare professionals should pay more attention to this group of patients on warfarin to achieve better outcomes.

## Background

Health-related quality of life (HRQoL) is a patient-reported outcome that enables healthcare professionals to understand patients’ perceptions of illnesses [1–3]. This metric is also used to measure the impact of disease and health interventions on overall health, covering various health dimensions, including the physical, psychological, social, and environmental dimensions [4]. Therefore, HRQoL is a humanistic outcome measure of overall health in patients with chronic diseases.

There are two types of HRQoL measurement instruments used to assess the parameter, which are generic and condition-specific instruments. Generic instruments are designed to assess the HRQoL level in a wide range of populations to enable HRQoL comparisons for both the general population and condition-specific patients. Among generic instruments, either health profile scores for each health dimension or a single index score, known as a “health utility score,” is adopted to report the HRQoL level. Nevertheless, its sensitivity to clinical changes for some condition-specific patients is diminished [5]. As a result, condition-specific instruments are developed to assess the HRQoL level in a specific patient group to enhance sensitivity to clinical changes; however, the HRQoL comparison across diverse populations is also limited [6, 7].

Cardiovascular diseases (CVDs) constitute a major health problem and the leading cause of death worldwide [8]. According to statistics from the Division of Noncommunicable Disease in Thailand, there are approximately 67,528 deaths due to CVDs each year, making CVDs the leading cause of death in Thailand [9]. Moreover, CVDs can affect a patient’s HRQoL because of the body function impairments they cause, and they require prolonged anticoagulant treatment [10, 11].

Warfarin is an anticoagulant used to treat and prevent thromboembolism for several CVDs, including atrial fibrillation, mechanical valve replacement, deep vein thrombosis, and pulmonary embolism [12]. However, it can cause several health concerns to both patients and healthcare professionals due to its complex regimens, high intrapatient variability, frequent blood monitoring for the international normalized ratio (INR), the occurrence of drug and herbal interactions, and the high associated incidence of internal bleeding [13]. As a result, prolonged warfarin therapy can potentially change the daily lifestyles of patients and also require dietary restrictions regarding herbs and vitamin K consumption since it can affect the therapeutic effect and alter the INR, resulting in diminished HRQoL. Moreover, patients may require lifelong anticoagulant therapy and regular follow-up at the hospital.

Previous studies have employed the World Health Organization Quality of Life Brief (WHOQOL-BREF) to assess HRQoL [4, 14]; however, condition-specific questionnaires for patients on warfarin, such as the Duke Anticoagulant Satisfaction Scale, are available [15, 16]. The 26-item WHOQOL-BREF was developed to assess a wide range of four health dimensions, including the physical, psychological, social, and environmental health dimensions; thus, it is practical and is currently widely used in clinical trials and clinical studies across the globe because more comprehensive information in terms of HRQoL scores for each health dimension is expected. A Thai-language version of the WHOQOL-BREF is also available [17]. Previous results from univariate analyses have shown that some sociodemographic factors, including age, sex, education level, and marital status, could affect the HRQoL levels for some health dimensions of the WHOQOL-BREF, whereas comorbidities other than CVDs could negatively affect patients’ HRQoL for all four health dimensions of patients on warfarin in both Malaysia and Pakistan [4, 14].

The willingness to pay (WTP) is a tool for estimating the related financial cost of a given condition. It asks patients how much they are willing to spend on therapy to treat or enhance their medical condition [18]. WTP can be measured through interviews and questionnaires. It is useful in assessing the burden of disease. It can also be used to assess the benefit of treatment, allocate resources for patient care, and be useful at the macro level, such as in the establishment of health policies, health economics, and the allocation of health services [19, 20]. Thus, the WTP is employed to determine whether patients with chronic diseases perceive medical therapy as being beneficial.

According to our literature review, there has been no published study conducted to assess the HRQoL and WTP among patients on warfarin in Thailand so far. Moreover, none of the condition-specific questionnaires have been translated and validated for HRQoL assessment among patients on warfarin in Thailand. To assess this parameter, we employed Thai WHOQOL-BREF, and EQ-5D-5L, which are both considered generic instruments, to provide the health profile and utility index scores for Thai patients on warfarin. Therefore, this study aimed to assess the HRQoL and WTP and identify the sociodemographic factors affecting both outcomes among patients on warfarin in Thailand.

## Methods

### Study design

This cross-sectional study was conducted on patients receiving warfarin therapy attending an outpatient anticoagulant clinic at three public hospitals in Thailand between June 2022 and June 2023. These included Chonburi Hospital (n = 159), Burapha University Hospital (n = 35), and Bang Lamung Hospital (n = 66). The inclusion criteria were age ≥ 18 years, reception of warfarin therapy due to any clinical indication for at least two months, ability to understand Thai, and provision of informed consent to be interviewed. Patients with life-threatening acute diseases, cognitive impairment, and disability were excluded from this study.

### Data collection

A purposive sampling method was employed to recruit eligible patients on warfarin therapy attending an outpatient anticoagulant clinic identified from patients’ medical records at three public hospitals in Thailand. Written informed consent was obtained from all eligible patients before their inclusion in this study. A face-to-face interview was conducted with patients while waiting to see their physicians. The research protocol was approved by Burapha University’s Institutional Review Board (IRB1-021/2566 Amendment 1) and Chonburi Hospital’s Institutional Review Board (37/65/O/q).

### Sample size calculation

The size of our study sample was calculated using this formula:

$$\mathrm{n}=\frac{{\left({\mathrm{Z}}_{1-\upbeta }\right)}^{2}\left[\mathrm{p}\left(1-\mathrm{p}\right)\right]}{{\mathrm{d}}^{2}}$$

where n is the required sample size, Z_1-β_ is Z value at power 1-β in which Z value is 1.64 at power 95%, p is the prevalence of patients receiving warfarin therapy in Thailand (40% per a previous study [21]), and d is margin of error (ideal value = 0.05). Our calculations yielded a minimum adequate sample size of 260 patients.

### Instruments

#### WHOQOL-BREF

Permission to use the WHOQOL-BREF was granted by the Director of the Suan Prung Psychiatric Hospital in Thailand [22]. The WHOQOL-BREF was proven to be a valid, reliable, and practical instrument for assessing HRQoL in the general Thai population and patient groups [22–25]. Our respondents were required to rate their health status during the past two weeks. It consisted of 24 items further categorized into four dimensions: physical health (7 items), psychological health (6 items), social relationships (3 items), and environment (8 items). Moreover, two other items for general health and overall quality of life were added, giving a total of 26 items. Each item has response options on a five-point Likert scale, including 1 (not at all), 2 (not much), 3 (moderately), 4 (a great deal), and 5 (completely). There are three negatively worded items (items 2, 9, and 11) whose scores should be reversed. The summation of raw scores from each score item within each health domain was transformed on a scale from 0 to 100 to enable HRQoL score comparisons among four health domains composed of different numbers of items. Higher scores indicate a better health status.

#### EQ-5D-5L

The Thai EQ-5D-5L was developed and permitted to be used by the EuroQoL group. It has two sections, which are a descriptive system and a visual analog scale (EQ-VAS). The descriptive system has five items for each of the following dimensions: mobility (MO), self-care (SC), usual activities (UA), pain/discomfort (PD), and anxiety/depression (AD). Each dimension has five response options, including no problem, slight problem, moderate problem, severe problem, and extreme problem/unable to perform. The responses to the five dimensions can be converted to a single score, the EQ-5D-5L index, using a country-specific value set. The Thai EQ-5D-5L index can range from ˗0.4212 to 1.000 [26]. Regarding the EQ-VAS, it is a self-rated health score on a straight line with two endpoints, 0 (the worst health imaginable state) and 100 (the best health imaginable state), yielding EQ-VAS scores ranging from 0 to 100 [27].

#### Willingness to pay

The WTP was assessed using a bidding game technique. Questions were asked to determine the amount of money the participants were willing to spend on warfarin therapy to manage or treat the patient’s illness. The questions about how much participants were willing to spend at once to visit the doctor were used. The patients were asked about the amount of money they were willing to spend. The possible answers for single payments were up to 8.75 USD (300 THB), up to 14.59 USD (500 THB), up to 23.34 USD (800 THB), up to 29.17 USD (1000 THB), up to 35.01 USD (1200 THB), up to 43.76 USD (1500 THB), up to 52.51 USD (1800 THB), up to 58.34 USD (2000 THB), and up to 72.93 USD (2500 THB) based on previous studies conducted in Thailand [28–30]. In addition, the patients were asked about the highest amount they could spend at once to visit the doctor. Furthermore, each participant was asked the following two additional questions: How often would you like to see your physician for warfarin therapy? Could you specify your additional medical conditions requiring attention from your physician? The WTP was converted from THB to USD using the exchange rate at the time (34.68 THB to 1 USD).

### Data analyses

Responses to two additional questions on warfarin therapy (frequencies of reception of warfarin therapy and additional medical conditions requiring a physician’s attention) were summarized using frequencies and percentages.

The sociodemographic and disease characteristics of patients were reported using descriptive statistics, including the mean, standard deviation, median, interquartile range (IQR), frequency, and percentage where appropriate. In addition, frequencies, and percentages were also used to report the responses to the WHOQOL-BREF and EQ-5D-5L items. Since four WHOQOL-BREF domain scores were normally distributed (Shapiro–Wilk test, p > 0.05), univariate analyses with an independent-sample t-test was used to determine the significant differences in HRQoL across sociodemographic and disease characteristic subgroups. Conversely, the EQ-5D index, EQ-VAS scores, and WTP were non-normally distributed (Shapiro–Wilk test, p < 0.05); therefore, the nonparametric Mann–Whitney U test was used to compare those scores across predefined subgroups. Then, multivariable linear regression was employed to investigate the associations between the significant sociodemographic factors identified from the univariate analysis as independent variables and the WHOQOL-BREF domain scores as the dependent variable. However, the associations between the EQ-5D index, EQ-VAS scores, and WTP as dependent variables and the significant sociodemographic factors as independent variables were investigated using a generalized linear model (GLM) with Gamma distribution and the log link model because it can accommodate the skewness and heteroscedasticity of those scores’ distributions [31, 32]. All analyses were performed using STATA 17 (StataCorp LLC, College Station, TX, USA), with a p-value of < 0.05 being considered statistically significant.

## Results

### Patient characteristics

Table 1 shows the sociodemographic and disease characteristics of all 260 patients. The mean age of our study participants was 58.3** ± **12.6 years, with an equal number of male and female patients recruited. However, the majority of patients (n = 161, 61.9%) were married and had no more than primary education (n = 158, 60.8%). Regarding disease characteristics, most patients received warfarin for valve replacement (n = 167, 64.2%) and reported having comorbidities other than CVDs (n = 178, 68.5%). The INRs of some of our participants (n = 138, 53.1%) were also out of the target range (2–3).

**Table 1:** Demographic information of study samples

Characteristics	Values
Gender, n (%)	
Male	130 (50.0)
Female	130 (50.0)
Age, mean ± SD	58.3 ± 12.6
Median (IQR)	60 (18)
Marital status, n(%)	
Single	62 (23.9)
Married	161 (61.9)
Widow	19 (7.3)
Divorced/Separated	18 (6.9)
Education level, n(%)	
Primary school or lower	158 (60.8)
Secondary school	71 (27.3)
College’s degree	11 (4.2)
Bachelor’s degree	17 (6.5)
Master’s degree or higher	3 (1.2)
Work status, n(%)	
Job/Business	141 (54.2)
Not working	119 (45.8)
Income, mean ± SD	10,831.7 ± 15,043.2
Median (IQR)	8000 (14,400)
Health insurance, n(%)	
Civil Servants Medical Benefits Scheme	13 (5.0)
Universal coverage	198 (76.2)
Social security scheme	43 (16.5)
Others	6 (2.3)
Warfarin indications, n(%)	
Atrial fibrillation	83 (31.9)
Valve replacements	167 (64.2)
Stroke	8 (3.1)
Deep vein thrombosis	10 (3.9)
Pulmonary embolism	1 (0.4)
Others	11 (4.2)
Comorbidities, n(%)	
No	82 (31.5)
Yes	178 (68.5)
Smoking status, n(%)	
Smokers	17 (6.5)
Non-smokers	243 (93.5)
Alcohol consumption, n (%)	
Drinkers	33 (12.7)
Non-drinkers	227 (87.3)
INR, n(%)	
Within target range (2–3)	122 (46.9)
Out of target range	138 (53.1)
Warfarin durations, n(%)	
< 1 year	34 (13.1)
≥ 1 year	226 (86.9)

### EQ-5D indexes, EQ-VAS, and WHOQOL-BREF scores

Table 2 presents the response distribution of EQ-5D-5L dimensions, EQ-5D indexes, and EQ-VAS scores. Patients on warfarin reported having problems (levels 2–5) with the highest percentage pertaining to PD (44.6%), followed by MO (40.4%), AD (18.8%), UA (18.5%), and SC (10.4%) having the lowest percentage. Furthermore, the mean EQ-5D index, and EQ-VAS score were 0.89** ± **0.15 and 76.92** ± **15.95, respectively. The percentages of ceiling effects were 34.2 for the EQ-5D index and 10.0 for the EQ-VAS score. In contrast, no floor effects were observed from the EQ-5D index and EQ-VAS score.

**Table 2:** Descriptive statistics of the Thai EQ-5D-5L’s responses, EQ-5D index, EQ-VAS scores and willingness to pay

Dimensions	Response distribution, n (%)				
	No problem	Slight problem	Moderate problems	Severe problem	Extreme problem/unable to perform
Mobility	155 (59.6)	47 (18.1)	42 (16.2)	13 (5.0)	3 (1.2)
Self-care	233 (89.6)	19 (7.3)	5 (1.9)	1 (0.4)	2 (0.8)
Usual activities	212 (81.5)	25 (9.6)	15 (5.8)	3 (1.2)	5 (1.9)
Pain/discomfort	144 (55.4)	81 (31.2)	27 (10.4)	5 (1.9)	3 (1.2)
Anxiety/depression	211 (81.2)	37 (14.2)	7 (2.7)	4 (1.5)	1 (0.4)

	Mean	SD	Range	% Ceiling	% Floor
EQ-5D index	0.89	0.15	− 0.024–1.000	34.2	0
EQ-VAS	76.92	15.95	25–100	10.0	0
Willingness to pay	22.25	32.19	0–291.76	N/A	N/A

*N/A* Non assessment

Table 3 presents responses to the Thai WHOQOL-BREF items. Most patients provided the “moderately” response to WHOQOL-BREF items for both physical and psychological health domains, except for Q24 (mobility), Q6 (concentration), Q7 (self-esteem), and Q23 (personal belief), whereas provided the “a great deal of” response for most WHOQOL-BREF items for both social and environmental health domains. It should be noted that three negatively worded items received the “moderately” response for both Q2 (pain) and Q11 (dependence on medical aids); however, Q9 (negative feeling) got the “not at all” response.

**Table 3:** Descriptive statistics of the Thai WHOQOL-BREF’s responses and four domain scores and utility scores derived from the Thai WHOQOL-BREF

WHOQOL-BREF items	Response distribution, n (%)				
	Not at all	Not much	Moderately	A great deal of	Completely
**Overall QoL & Health**					
Satisfied with health (Q1)	5 (1.9)	18 (6.9)	142 (54.6)	80 (30.8)	15 (5.8)
Quality of life rating (Q26)	1 (0.4)	9 (3.5)	137 (52.7)	80 (30.8)	33 (12.7)
**Physical health**					
Pain (Q2)	70 (26.9)	68 (26.2)	90 (34.6)	28 (10.8)	4 (1.5)
Energy (Q3)	11 (4.2)	19 (7.3)	111 (42.7)	103 (39.6)	16 (6.2)
Sleep (Q4)	26 (10.0)	30 (11.5)	89 (34.2)	80 (30.8)	35 (13.5)
Activities of daily living (Q10)	1 (0.4)	12 (4.6)	107 (41.2)	106 (40.8)	34 (13.1)
Dependence on medical aids (Q11)	20 (7.7)	17 (6.5)	101 (38.9)	86 (33.1)	36 (13.9)
Work capacity (Q12)	14 (5.4)	17 (6.5)	105 (40.4)	102 (39.2)	22 (8.5)
Mobility (Q24)	14 (5.4)	24 (9.2)	62 (23.9)	107 (41.2)	53 (20.4)
**Psychological health**					
Positive feeling (Q5)	6 (2.3)	6 (2.3)	123 (47.3)	97 (37.3)	28 (10.8)
Concentration (Q6)	4 (1.5)	17 (6.5)	94 (36.2)	108 (41.5)	37 (14.2)
Self-esteem (Q7)	2 (0.8)	11 (4.2)	104 (40.0)	111 (42.7)	32 (12.3)
Bodily-image (Q8)	0	8 (3.1)	116 (44.6)	93 (35.8)	43 (16.5)
Negative feeling (Q9)	143 (55.0)	39 (15.0)	59 (22.7)	18 (6.9)	1 (0.4)
Personal belief (Q23)	1 (0.4)	11 (4.2)	80 (30.8)	111 (42.7)	57 (21.9)
**Social health**					
Personal relationship (Q13)	5 (1.9)	14 (5.4)	91 (35.0)	116 (44.6)	34 (13.1)
Social support (Q14)	12 (4.6)	17 (6.5)	92 (35.4)	107 (41.2)	32 (12.3)
Sexual activity (Q25)	44 (16.9)	32 (12.3)	98 (37.7)	63 (24.2)	23 (8.9)
**Environment**					
Security (Q15)	6 (2.3)	12 (4.6)	84 (32.3)	122 (46.9)	36 (13.9)
Home environment (Q16)	1 (0.4)	7 (2.7)	74 (28.5)	137 (52.7)	41 (15.8)
Financial support (Q17)	11 (4.2)	26 (10.0)	129 (49.6)	72 (27.7)	22 (8.5)
Health care (Q18)	6 (2.3)	9 (3.5)	93 (35.8)	117 (45.0)	35 (13.5)
Accessibility of needed information (Q19)	1 (0.4)	22 (8.5)	101 (38.9)	105 (40.4)	31 (11.9)
Leisure activity (Q20)	22 (8.5)	17 (6.5)	99 (38.1)	91 (35.0)	31 (11.9)
Physical environment (Q21)	3 (1.2)	11 (4.2)	92 (35.4)	118 (45.4)	36 (13.9)
Transport (Q22)	10 (3.9)	28 (10.8)	95 (36.5)	100 (38.5)	27 (10.4)

	Mean	SD	Range	% Ceiling	% Floor
**Four domain scores**					
Physical	59.18	14.13	19–100	0.38	0
Psychological	68.56	15.47	25–100	3.08	0
Social	59.13	19.64	0–100	3.08	0.38
Environmental	65.23	14.04	25–100	2.31	0

Table 3 also presents the average four WHOQOL-BREF domain scores. The highest mean WHOQOL-BREF domain score was psychological health (68.56** ± **15.47) followed by environmental health (65.23** ± **14.04), physical health (59.18** ± **14.13), and social health (59.13** ± **19.64). It was also revealed that both psychological and social health domains yielded the highest ceiling effects (3.08%), followed by environmental health (2.31%) and physical health (0.38%). Most of the WHOQOL-BREF domains did not show floor effects, except for social health with a value of 0.38%.

### Association between the EQ-5D indexes, EQ-VAS and WHOQOL-BREF domain scores, and sociodemographic, and disease characteristics

Table 4 shows the univariate analyses performed between sociodemographic and disease characteristics and the EQ-5D indexes, EQ-VAS, and WHOQOL-BREF domain scores. Per these analyses, age, marital status, education level, work status, income, comorbidities, alcohol consumption, INR level, and warfarin duration were the significant factors affecting the HRQoL. We also found that age and comorbidities affected most of the HRQoL scores obtained from EQ-5D-5L and WHOQOL-BREF. Older patients (> 60 years) produced lower EQ-5D indexes, EQ-VAS, and WHOQOL-BREF domain scores than younger patients (≤ 60 years; all p < 0.05) except for the environmental health domain (p = 0.3183). Similarly, patients with comorbidities had lower HRQoL scores than those without comorbidities (p < 0.05), except for EQ-VAS (p = 0.0599) and the environmental health domain (p = 0.1406).

**Table 4:** Comparison of EQ-5D index, EQ-VAS, WHOQOL-BREF domain and willingness to pay mean scores, standard deviations and significant levels based on the samples’ demographic information

Demographic factors	EQ-5D-5L		WHOQOL-BREF				Willingness to pay (USD)
	EQ-5D index	EQ-VAS	Physical	Psychological	Social	Environmental	
**Gender**							
Male	0.89 ± 0.17	77.04 ± 14.48	60.65 ± 14.15	68.55 ± 15.43	60.22 ± 18.50	65.05 ± 13.91	25.15 ± 35.81
Female	0.88 ± 0.14	76.81 ± 17.35	57.71 ± 14.01	68.57 ± 15.58	58.04 ± 20.73	65.41 ± 14.21	19.35 ± 27.93
*p* value	0.0665	0.7091	0.0937	0.9904	0.3709	0.8394	0.3302
**Age (years) grouped by median**							
≤ 60	0.91 ± 0.12	79.57 ± 15.31	62.60 ± 14.26	71.07 ± 13.84	63.90 ± 17.75	66.07 ± 13.16	23.75 ± 32.65
> 60	0.86 ± 0.18	74.11 ± 16.19	55.54 ± 13.10	65.88 ± 16.68	54.06 ± 20.34	64.33 ± 14.91	20.65 ± 31.73
*p* value	**0.0059**	**0.0037**	** < 0.0001**	**0.0069**	** < 0.0001**	0.3183	**0.0490**
**Marital status**							
Single/Separated/ Divorced/Widow	0.89 ± 0.12	76.24 ± 16.73	58.18 ± 13.41	67.33 ± 15.54	58.09 ± 19.22	62.12 ± 13.65	18.62 ± 21.57
Married	0.88 ± 0.17	77.34 ± 15.49	59.79 ± 14.56	69.31 ± 15.43	59.77 ± 19.93	67.14 ± 13.97	24.48 ± 37.13
*p* value	0.5333	0.6810	0.3743	0.3180	0.5043	**0.0049**	0.0678
**Education level**							
Primary or secondary	0.89 ± 0.14	76.79 ± 16.24	58.55 ± 13.94	68.06 ± 15.26	58.24 ± 19.77	64.38 ± 13.45	21.93 ± 32.81
Higher secondary and above	0.89 ± 0.22	77.87 ± 13.84	63.84 ± 14.88	72.26 ± 16.74	65.74 ± 17.62	71.48 ± 16.75	24.59 ± 27.50
*p* value	0.0506	0.9389	0.0501	0.1564	**0.0456**	**0.0080**	0.1859
**Work status**							
Job/Business	0.91 ± 0.12	78.76 ± 14.43	61.02 ± 13.05	69.16 ± 13.90	61.71 ± 16.65	65.20 ± 13.29	24.31 ± 32.15
Not working	0.86 ± 0.18	74.75 ± 17.39	56.99 ± 15.08	67.84 ± 17.19	56.08 ± 22.37	65.27 ± 14.93	18.41 ± 32.06
*p* value	**0.0039**	0.0700	**0.0217**	0.5010	**0.0244**	0.9680	**0.0327**
**Income (THB) grouped by median**							
≤ 8,000	0.87 ± 0.14	73.71 ± 17.56	55.49 ± 13.73	67.40 ± 15.37	57.52 ± 20.72	63.32 ± 14.25	21.21 ± 37.61
> 8000	0.90 ± 0.16	79.94 ± 13.66	62.64 ± 13.67	69.65 ± 15.55	60.65 ± 18.52	67.03 ± 13.64	23.52 ± 24.06
*p* value	**0.0009**	**0.0080**	** < 0.0001**	0.2415	0.1992	**0.0328**	**0.0055**
**Warfarin indication**							
AF/Valve replacements	0.89 ± 0.14	77.25 ± 16.04	59.14 ± 13.66	68.53 ± 15.20	59.23 ± 19.46	65.08 ± 13.88	21.55 ± 31.87
DVT/PEs	0.82 ± 0.24	73.41 ± 14.83	59.55 ± 18.86	68.82 ± 18.55	58.05 ± 21.94	66.86 ± 15.83	27.35 ± 30.63
*p* value	0.1839	0.1603	0.9230	0.9344	0.7871	0.5694	0.9973
**Comorbidities**							
No	0.94 ± 0.09	79.79 ± 14.80	64.85 ± 13.42	73.15 ± 14.39	65.63 ± 19.41	67.12 ± 12.83	29.61 ± 39.24
Yes	0.86 ± 0.17	75.60 ± 16.32	56.56 ± 13.71	66.44 ± 15.54	56.13 ± 19.07	64.36 ± 14.51	18.55 ± 27.36
*p* value	**0.0001**	0.0599	** < 0.0001**	**0.0011**	**0.0003**	0.1406	**0.0011**
**Smoking**							
Non-smokers	0.89 ± 0.16	77.18 ± 15.60	58.89 ± 13.91	68.61 ± 15.09	59.33 ± 19.26	65.31 ± 13.67	23.25 ± 34.96
Smokers	0.93 ± 0.09	73.24 ± 20.54	63.29 ± 16.96	67.76 ± 20.78	56.24 ± 25.02	64.12 ± 19.00	18.51 ± 18.23
*p* value	0.3999	0.5499	0.2147	0.8706	0.5306	0.8026	0.4217
**Alcohol consumptions**							
Non-drinkers	0.88 ± 0.16	76.76 ± 16.11	58.36 ± 14.02	68.60 ± 15.21	59.08 ± 19.50	65.12 ± 13.91	21.80 ± 33.56
Drinkers	0.96 ± 0.07	78.03 ± 14.94	64.82 ± 13.83	68.27 ± 17.42	59.45 ± 20.90	66.00 ± 15.06	23.35 ± 28.69
*p* value	**0.0003**	0.7432	**0.0138**	0.9101	0.9195	0.7369	0.8649
**INR level**							
Within target range (2–3)	0.90 ± 0.14	78.98 ± 16.06	60.43 ± 13.99	68.07 ± 14.99	59.49 ± 20.01	65.02 ± 14.68	18.49 ± 16.74
Out of target range	0.88 ± 0.17	75.10 ± 15.68	58.07 ± 14.21	68.99 ± 15.93	58.81 ± 19.38	65.41 ± 13.49	25.57 ± 41.07
*p* value	0.4168	**0.0316**	0.1778	0.6363	0.7811	0.8242	0.5170
**Warfarin duration**							
< 1 year	0.85 ± 0.18	70.44 ± 17.34	54.21 ± 15.80	68.38 ± 17.85	57.41 ± 20.82	65.15 ± 12.82	34.10 ± 53.55
≥ 1 year	0.89 ± 0.15	77.90 ± 15.54	59.92 ± 13.75	68.58 ± 15.13	59.39 ± 19.49	65.24 ± 14.23	20.46 ± 27.34
*p* value	0.0783	**0.0195**	**0.0275**	0.9437	0.5851	0.9703	0.1001

Bold values indicate significant at* p* < 0.05

*AF* Atrial Fibrillations *DVT* Deep vein thrombosis *PE* Pulmonary embolisms

Table 5 presents the GLM of demographic and disease characteristics and HRQoL scores. After the significant factors identified from the univariate analysis were entered in the GLM, it revealed that older patients (> 60 years) had lower physical health domain scores [β =  − 3.719, p = 0.041] and social health domain scores [β =  − 6.424, p = 0.013] than younger patients (≤ 60 years). Patients with comorbidities had lower WHOQOL-BREF domain scores for physical health [β =  − 5.613, p = 0.0047], psychological health [β =  − 5.390, p = 0.013], social health [β =  − 6.669, p = 0.015], and the EQ-5D index [β =  − 0.557, p = 0.025] than their counterparts. Moreover, higher environmental health scores were observed in married [β = 4.309, p = 0.016], and highly educated patients [β =  5.795, p = 0.031] compared to unmarried and less educated patients; meanwhile, patients with higher income and those who received warfarin therapy for more than a year had higher EQ-VAS scores than their counterparts after adjusting for other sociodemographic and disease factors.

**Table 5:** Generalized linear model of demographic factors associated with the EQ-5D index, EQ-VAS, WHOQOL-BREF domain scores and willingness to pay

Sample characteristics	WHOQOL-BREF domains^a^								EQ-5D index^b^		EQ-VAS^b^		Willingness to pay^b^	
	Physical health		Psychological health		Social health		Environmental health							
	Coefficients	*P*-value	Coefficients	*P*-value	Coefficients	*P*-value	Coefficients	*P*-value	Coefficients	*P*-value	Coefficients	*P*-value	Coefficients	*P*-value
**Age (years) grouped by median (Ref.: ≤ 60)**														
> 60	− 3.719	0.041	− 3.389	0.093	− 6.424	0.013	N/A	N/A	− 0.034	0.149	− 0.046	0.083	− 1.34	0.732
**Marital status (Ref.: Single/Separated/Divorced/Widow)**														
Married	N/A	N/A	N/A	N/A	N/A	N/A	4.309	0.016	N/A	N/A	N/A	N/A	N/A	N/A
**Education level (Ref.: Primary or secondary)**														
Higher secondary and above	N/A	N/A	N/A	N/A	6.641	0.069	5.795	0.031	N/A	N/A	N/A	N/A	N/A	N/A
**Work status (Ref.: Not working)**														
Job/Business	− 0.795	0.675	N/A	N/A	2.694	0.269	N/A	N/A	0.038	0.126	N/A	N/A	− 0.87	0.830
**Income (THB) grouped by median (Ref: ≤ 8,000)**														
> 8000	5.116	0.007	N/A	N/A	N/A	N/A	2.398	0.171	− 0.007	0.770	0.067	0.010	0.73	0.851
**Comorbidities (Ref: No)**														
Yes	− 5.613	0.004	− 5.390	0.013	− 6.669	0.015	N/A	N/A	− 0.557	0.025	N/A	N/A	− 11.72	0.029
**Alcohol consumptions (Ref: Non-drinkers)**														
Drinkers	4.555	0.068	N/A	N/A	N/A	N/A	N/A	N/A	0.074	0.022	N/A	N/A	N/A	N/A
**INR levels (Ref.: Out of target range)**														
Within target range (2–3)	N/A	N/A	N/A	N/A	N/A	N/A	N/A	N/A	N/A	N/A	-0.043	0.094	N/A	N/A
**Warfarin durations (Ref.: < 1 year)**														
≥ 1 year	4.699	0.054	N/A	N/A	N/A	N/A	N/A	N/A	N/A	N/A	0.081	0.032	N/A	N/A

*N/A* Non-assessment

^a^Multivariable linear regression analysis

^b^Generalized linear model with Gamma distribution and log link function

### WTP

The mean WTP was 22.25** ± **32.19 USD for one patient’s visit. Ninety percent of our participants were willing to pay for their warfarin treatment. A significant association between WTP values and some sociodemographic and disease characteristics was observed; these included age, work status, income, and comorbidities. Patients aged under 60 years demonstrated a significantly greater WTP than patients aged at least 60 years (p = 0.0490). Higher WTP values were observed in patients who had a job or business (p = 0.0327), higher income (p = 0.0055), and no comorbidities (p = 0.0011). WTP mean scores, standard deviations, and significant levels are presented in Table 4.

The GLM of demographic and disease characteristics and WTP is shown in Table 5, where statistically significant factors from the univariate analysis were entered in the GLM. The result showed that patients with comorbidities had lower WTP [β =  − 11.72, p = 0.029] than those without comorbidities.

The majority of patients (n = 94, 37.45%) indicated that the most appropriate follow-up interval was every 12 weeks (3 months), and they also mentioned that there were no further medical conditions requiring attention from their physicians (n = 88, 73.95%).

## Discussion

To the best of our knowledge, this is the first study to assess the HRQoL and WTP alongside identifying some significant sociodemographic and disease factors affecting those two outcomes among patients on warfarin in Thailand.

As expected, our study showed that patients on warfarin had a lower mean EQ-5D index (0.89** ± **0.15) than the general Thai population (0.93** ± **0.10) [33]. Our results also revealed that the ceiling effect of the EQ-5D index (34.2%) was lower than that of the general Thai population (49.1%) [34], implying that the CVDs could considerably diminish the HRQoL levels of patients on warfarin. Moreover, some patients on warfarin who presented with comorbidities other than CVD could also diminish the HRQoL level. Notably, the percentages of patients reporting “no problem” for each EQ-5D-5L dimension were as follows: SC (89.6%), UA (81.5%), AD (81.2%), MO (59.6%), and PD (55.4%). These percentages were lower than those of the general Thai population, yielding lower EQ-5D indexes. Nevertheless, this response distribution pattern was similar to the results of the general Thai population.

According to the Thai WHOQOL-BREF domain scores, the highest score was that of psychological health (68.56** ± **15.47), whereas the lowest score was that of social health (59.13** ± **19.64). Unlike the pattern WHOQOL-BREF domain scores of the two previous studies conducted in Malaysia and Pakistan [4, 14], both studies showed that social health had the second highest domain scores. This discrepancy could be explained by the cultural difference across countries and the fact that the social health domain has a specific question asking about patients’ sexual life, which could make the respondents feel shy and reluctant to respond (for Thai patients) [35], resulting in lower scores in the social health domain. Nevertheless, our results were in line with those of previous studies in which the psychological health domain produced the highest domain scores. A possible explanation is that respondents with cognitive impairment were excluded from this study, which means respondents were less likely to have psychological problems during their follow-up. In addition, we excluded respondents with some acute life-threatening diseases, implying that their disease conditions were quite stable, and no acute adverse events like bleeding due to warfarin therapy were observed; therefore, they may have adequate acceptance regarding their disease conditions without any negative feelings, resulting in the high psychological domain scores. Nevertheless, future studies should re-assess the HRQoL using a disease-specific questionnaire for patients on warfarin.

Although there were several demographic and disease factors affecting the HRQoL for patients on warfarin from the univariate analysis, the GLM yielded different results, possibly because generic instruments (EQ-5D-5L and WHOQOL-BREF) were employed to measure HRQoL scores due to their limited sensitivity to clinical changes. As a result, condition-specific instruments should be employed to reinvestigate these associations among patients on warfarin in future studies. The GLM revealed that the physical and social WHOQOL-BREF domain scores decreased with the advanced age of the respondents. Unlike a previous study [14], it did not show any significant associations with any WHOQOL-BREF domain scores. This was probably because the ages of our study participants varied significantly (18–92 years old), and the median age of our study participants (60 years; IQR, 49–67 years) was higher than that of the participants of the previous study; therefore, our study was more suitable for distinguishing the HRQoL domain scores of young respondents from those of old respondents than the previous study. Nevertheless, our finding is consistent with the findings of the previous study [14] which revealed that physical, psychological, and social WHOQOL-BREF domain scores were lower for respondents with comorbidities than their counterparts.

Similar to the HRQoL scores, the univariate analysis showed significant associations between WTP and some demographic factors including age, work status, income, and comorbidities. As expected, the GLM showed that patients with comorbidities had lower WTP than those without comorbidities. A previous study showed that WTP increased with the severity and probability of occurrence of comorbidities [36]. However, our result showed a different direction of the association between WTP and comorbidities. This could be because the patients were still not satisfied with the warfarin therapy since it could not improve their medical conditions as much as they would have wanted, especially as CVDs are more likely to worsen over time [37].

Some variations between WHOQOL-BREF domain scores and the EQ-5D indexes were observed concerning the association with sociodemographic and disease factors. Specifically, age was associated with physical and social WHOQOL-BREF domain scores, which was not the case for the EQ-5D index. A possible explanation is that age was associated with both physical and social WHOQOL-BREF domain scores, whereas the EQ-5D index was computed from the responses to the dimensions related to physical and psychological health. Therefore, the EQ-5D index did not show any significant association with the age of respondents. Conversely, the presence of comorbidities was significantly associated with both WHOQOL-BREF domain scores and the EQ-5D indexes because it was associated with both physical and psychological WHOQOL-BREF domains, which are the same dimensions used to compute the EQ-5D indexes.

The GLM also showed that there were variations in responses to the EQ-5D indexes and EQ-VAS scores. In line with the findings of a previous study [38], the monthly income was significantly associated with the EQ-VAS, which was not the case with the EQ-5D indexes. This was possibly because the respondents may conceptually rate some aspects through the EQ-VAS beyond the EQ-5D dimensions. Furthermore, an unexpected direction of the association between alcohol consumption and the EQ-5D indexes was detected because it revealed that alcohol drinkers had higher EQ-5D indexes than their counterparts. This aligns with the findings of previous general Thai population studies [33, 39] which showed that drinkers reported higher EQ-5D indexes and most SF-36v2 dimension scores, except for the social functioning dimension. It might be due to specific characteristics of Thai population, and this finding should be further investigated in future research.

In Thailand, there is an advanced, and specialized care clinic specific to patients on warfarin implemented in many Thai public hospitals where physicians, pharmacists, and nurses are providing essential care, patient education, medication, and diet counseling related to warfarin therapy. According to this current study’s findings, advanced age, and the presence of comorbidities could play a significant role in diminished HRQoL and WTP; therefore, healthcare professionals should pay more attention to these groups of patients on warfarin to achieve better patient-reported outcomes.

Several limitations should be addressed. First, the cross-sectional study design might not reflect the changes in HRQoL and WTP over time; therefore, a longitudinal study design should be employed in future studies. Second, both EQ-5D-5L, and WHOQOL-BREF are generic questionnaires that are not specific to patients on warfarin; therefore, future studies should assess the HRQoL using a disease-specific questionnaire which is more sensitive to health changes for patients on warfarin. Third, this study was mainly conducted in three hospitals in Thailand, which means it may not be adequately representative of patients on warfarin in Thailand. Fourth, this study did not assess the HRQoL of patients with some adverse events related to warfarin use such as uncontrolled INR or bleeding.

## Conclusions

As expected, our study demonstrated that patients on warfarin had lower EQ-5D indexes and EQ-VAS scores than the general Thai population [33]. Furthermore, the Thai patients on warfarin had lower four-domain WHOQOL-BREF scores than those reported in previous studies [4, 14]. The GLM demonstrated that advanced age (> 60 years) and the presence of comorbidities other than CVD are two significant factors diminishing the HRQoL of patients on warfarin; however, only the presence of comorbidities showed a significant association with WTP values. All healthcare professionals should therefore pay more attention to these groups of patients on warfarin.

## Data Availability

The data analyzed and reported in this manuscript is not available for public sharing because all raw data should be kept it with researcher for privacy in order to comply with the ethical standard.

## Abbreviations

AD: Anxiety/depression
CVDs: Cardiovascular diseases
EQ-5D: EuroQoL-5 dimensions
EQ-VAS: EuroQol visual analog scale
GLM: Generalized linear model
HRQoL: Health-related quality of life
INR: International normalized ratio
IQR: Interquartile range
PD: Pain/discomfort
SC: Self-care
UA: Usual activities
WHOQOL-BREF: World Health Organization Quality of Life Brief
WTP: Willingness to pay
